# Basal ganglia infarction and COVID-19 infection in an elderly patient: A case report

**DOI:** 10.1515/tnsci-2020-0194

**Published:** 2021-11-05

**Authors:** Manar Ahmed Kamal

**Affiliations:** Faculty of Medicine, Benha University, Fareed Nada Street, Benha City, Qalubiya Governorate, 13511, Egypt

**Keywords:** basal ganglia infarction, neurological manifestation, case report, COVID-19, hematological characters

## Abstract

**Background:**

Coronavirus disease 2019 (COVID-19) has spread rapidly worldwide since the first cases were observed in Wuhan, China. Patients with COVID-19 develop multiple neurological symptoms, including headache, disturbed consciousness, and paresthesia, in addition to systemic and respiratory symptoms.

**Case presentation:**

We presented a 57-year-old woman admitted to the emergency department – in December 2020 – with complaints of slurred speech, confusion, and left upper limb weakness after one week of positive nasopharyngeal swab sample SARS-CoV-2.

**Conclusions:**

While the patient had previous comorbidities like hypertension and diabetes, she had no prior history of ischemic stroke or thrombosis, so we conclude that unilateral acute basal ganglia infarction may be a unique neurological manifestation after COVID-19 infection in an elderly patient with previous comorbidities.

## 1 Background

Coronavirus disease 2019 (COVID-19) is a novel disease caused by severe acute respiratory syndrome coronavirus 2 (SARS-CoV-2). SARS-CoV-2 has spread rapidly worldwide since the first cases in Wuhan, China, were observed in December 2019 [1]. Patients with COVID-19 develop neurological symptoms, including headache, disturbed consciousness, and paresthesia [2], in addition to systemic and respiratory symptoms. Stroke is one of the most typical neurological manifestations associated with COVID-19 [3,4]. Also, basal ganglia hemorrhage [5,6] and altered mental status are neurological manifestations of coronavirus disease 2019 [5]. We describe a case of basal ganglia infarction associated with COVID-19 in a female elderly patient. The report is early published as a preprint in Research Square [7].

## 2 Case presentation

**Figure 1:**
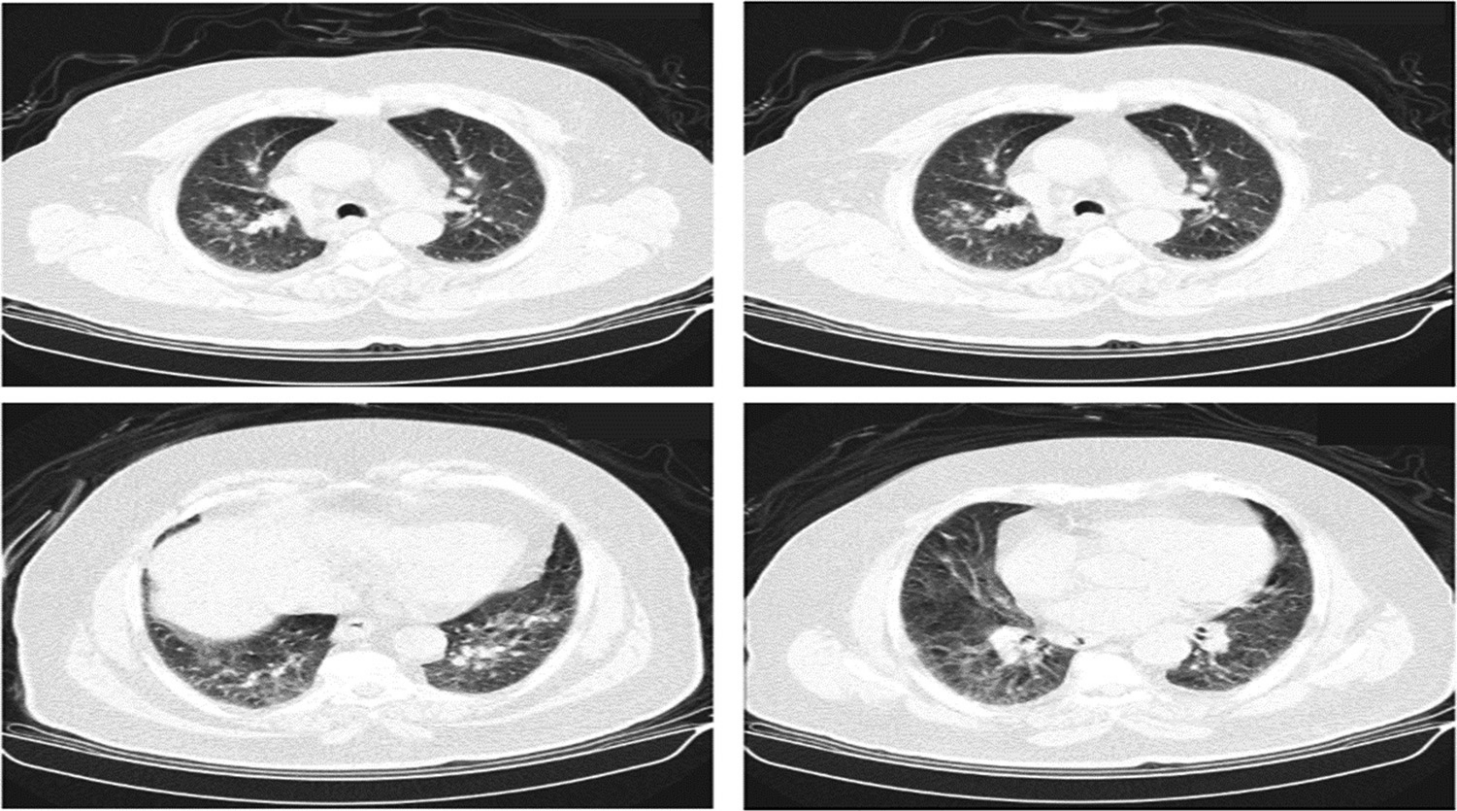
Computerized tomography (CT) on the lung. Multiple axial contiguous thin cuts were taken through the chest and have shown few right-side apical small ground-glass consolidation patches with bilateral mild subpleural lower lobar ground-glass haze more accentuated on the right side; in addition to mild to moderate cardiomegaly and right adrenal lesion with fat-rich adenoma.

A 57-year-old woman presented to the emergency department (ED) – in December 2020 – with complaints of slurred speech, confusion, and left upper limb weakness. Her medical history included suffering from a persistent fever, severe headache, cough, fatigue, anosmia, dysgeusia, sore throat, vomiting, dizziness, fatigue, and bony pain, and the reverse transcription-polymerase chain reaction (RT-PCR) assay of nasopharyngeal swab sample was positive for SARS-CoV-2 from one week before presentation in ED. The patient also has diabetes mellites and hypertension in her medical history. All routine diagnostic tests were done, and the patient’s blood analysis showed an increase in red blood cells (RBCs), lymphocytes count, a marked increase in C-reactive protein (CRP), and D-dimer due to infection. She had a slightly decreasing mean corpuscular hemoglobin concentration and a marked increase in fasting blood glucose (FBS) as diabetes (Table 1). The patient weight was 70 kg; height: 150 cm; body mass index (BMI): 31 kg/m^2^; and the blood pressure: 140/100 mm Hg sitting. The pulse was 90/min, and oxygen saturation was 90%. Chest computed tomography (CT) and magnetic resonance imaging (MRI) on the brain were done for the patient. CT of the lung showed few right-side apical small ground-glass consolidation patches with bilateral mild subpleural lower lobar ground-glass haze more accentuated on the right side that scientifically reefed to right-side viral pneumonia because of COVID-19. Few scattered sub-centimetric emphysematous bullae were noted with fine scattered subpleural atelectatic bands. Mediastinal structures are normal with a patent tracheobronchial tree. There is no mediastinal, hilar adenopathy, or pleural effusion (Figure 1). The scanned arterial tree, including the coronary vessels, involved advanced atherosclerotic changes and mild to moderate cardiac chamber enlargement. Visualized cuts of the upper abdomen revealed a well-defined right adrenal lesion with internal fat density measuring about 5.2 cm × 4 cm, primarily representing fat-rich adenoma with few bilateral simple cortical renal cysts (Figure 1).

**Table 1:** Laboratory tests and examinations

Laboratory tests	Patient-level	Normal level	Unit
Complete blood count (CBC)			
Hemoglobin (Hb)	13.2	12–16	g/dL
RBCs	5.39	3.8–4.8	×10^6^/UL
HCT	45.1	36–46	L/L
MCV	83.7	80–101	Fl
MCH	26.5	26–32	Pg
MCHC	29.3	31–34	g/dL
Platelet count	337	150–400	×10^3^/µL
WBCs	6.5	4–11	×10^3^/µL
Differential leucocyte count			
Neutrophils	45	40–80	%
1. Staff	3	0–8	%
2. Segmented	42	40–75	%
Lymphocytes	45	20–40	%
Monocytes	8	2–10	%
Eosinophils	2	1–6	%
Basophils	0	0–1	%
INR	1.02	1–3	
*Serum* creatinine	0.9	0.6–1.10	mg/dL
S.G.O.T (AST)	32	Up to 40	U/L
S.G.P.T (ALT)	38	Up to 40	U/L
FBS	238	99 or lower	mg/dL
CRP	31.7	below 3.0	mg/L
D-dimer	0.720	below 0.500	ng/mL
Ferritin	109.9	12–263	ng/mL

The patient’s blood analysis showed an increase in RBCs, lymphocyte count, a marked increase in CRP, and a slight increase of D-dimer due to infection. In addition, she had a slight decrease in mean corpuscular hemoglobin concentration and a significant rise in FBS as she has diabetes.

Abbreviations: RBCS: blood red blood cells (erythrocytes); HCT: hematocrit; MCV: mean corpuscular volume; MCH: mean corpuscular hemoglobin; MCHC: mean corpuscular hemoglobin concentration; MPV: mean platelet volume; WBCS: white blood cells; INR: the international normalized ratio; SGOT: serum glutamic oxaloacetic transaminase; AST: aspartate aminotransferase; SGPT: serum glutamic pyruvic transaminase; ALT: alanine aminotransferase; FBS: fasting blood glucose; CRP: C-reactive protein.

**Figure 2:**
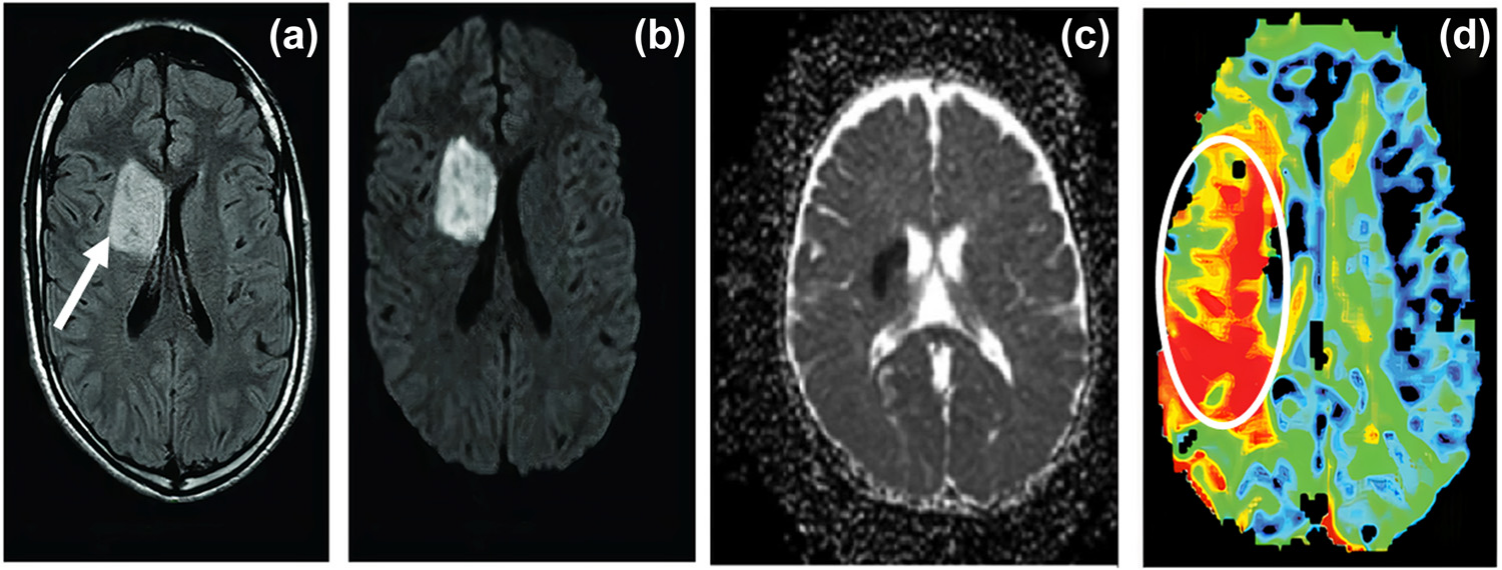
MRI on the brain. MRI on the brain has shown acute right basal ganglia infarct. (a) FLAIR, (b) DWI, (c) ADC, and (d) PWI.

Brain MRI showed acute infarction of the right basal ganglia (Figure 2) and abnormal hyperintense signal along with the right caudate head and anterior limb of the internal capsule in (a) fluid-attenuated inversion recovery (FLAIR) sequence (arrow) associated with positive mass effect on the right lateral ventricle. The corresponding area of restricted diffusion was appreciated as a hyperintense signal in (b) diffusion-weighted imaging (DWI) and hypointense signal in (c) apparent diffusion coefficient (ADC) denoting restricted distribution in the DWI and ADC map, (d) perfusion-weighted imaging (PWI) showing corresponding markedly reduced perfusion in the affected area (circle) impressive of right caudate head/anterior limb of internal capsule acute infarction. The images were imported into the viewing software (OsiriX Lite^®^).

Intravenous recombinant tissue plasminogen activator (rt-PA) was given to patients within 3 hours after onset. In addition to starting the COVID-19 therapeutic course, the decision was taken to admit the patient to an intensive care unit (ICU) until stabilizing O_2_ saturation. We treated the patient with intravenous ceftriaxone (2 g/day for 14 days), methylprednisolone (60 mg daily over six months), and anticoagulation over three months. Symptoms resolved entirely within 72 h. The patient was discharged after two weeks of antibiotic therapy. During a follow-up period of two weeks, no new symptoms occurred, and the second nasopharyngeal swab by RT-PCR assay was negative for SARS-CoV-2.


**Informed consent:** Informed consent has been obtained from all individuals included in this study.
**Ethical approval:** The research related to human use has been complied with all the relevant national regulations, institutional policies, and in accordance with the tenets of the Helsinki Declaration and has been approved by the Research Ethical Committee (REC) in the Faculty of Medicine – Benha University, Egypt (Ethical Approval Number: RC.10.1.2021).

## 3 Discussion

Basal ganglia infarction is a rare type of cerebral infarct with unique clinical manifestations [8]. As in this report, many factors may lead to basal ganglia infarction, including diabetes mellites [9] and recently COVID-19. Among patients with diabetes, the risk of vascular events is significantly increased compared to nondiabetics [9]. The patient was a confirmed case of SARS-CoV-2 Infection, which agrees with the evidence that elderly patients are more susceptible to infection [10]. Our patient developed right-sided viral pneumonia, a significant cause of death in patients with cerebral infarction. Nakagawa and colleagues showed that the pneumonia mortality rate in patients with basal ganglia infarcts was significantly higher than in patients with or without cerebral hemispheric strokes in other locations [11]. Due to right-sided viral pneumonia, the patient was admitted to ICU to protect her life. There is ample evidence that COVID-19 may be associated with many neurological conditions [4] such as stroke [3], facial nerve palsy [12], Guillain-Barré syndrome [13], and basal ganglia hemorrhage [5,6]. A case series conducted in 2020 on 3,556 hospitalized patients with a diagnosis of COVID-19 infection showed that the incidence of ischemic stroke in COVID-19 patients was relatively lowered; 32 patients (0.9%) had imaging proven ischemic stroke [14], while Tan and colleagues report that the pooled incidence of acute ischemic stroke (AIS) in COVID-19 patients was about 1.2%, with a high mortality rate [3]. However, the underlying stroke mechanism of COVID-19 remains debatable [3]. Elevated D-dimer is prominent in COVID-19 patients with concomitant ischemic stroke, but further mechanistic studies are required to elucidate their role in the pathogenesis of AIS. However, multiple studies described neurological complications of COVID-19; no previous evidence presented the association between basal ganglia infarction and COVID-19 infection.

## 4 Conclusions

While the patient had previous comorbidities like hypertension and diabetes, she had no prior history of ischemic stroke or thrombosis, so we conclude that unilateral acute basal ganglia infarction may be a unique neurological manifestation after COVID-19 infection in an elderly patient with previous comorbidities. The most predicted mechanism depends mainly on D-dimer changes. The learned Lesson of this case report is the rapid bringing of the patient if they have any of the following symptoms (FAST): F: Facial drooping, A: Arm weakness, S: Speech difficulties, and T: Time to call for an emergency. The learned Lesson for doctors is accurate medical history-taking and rapid.

## List of abbreviations

COVID-19: coronavirus disease 2019
ED: emergency department
SARS-CoV-2: severe acute respiratory syndrome coronavirus 2
RT-PCR: reverse transcription-polymerase chain reaction
RBCs: red blood cells
CRP: C-reactive protein
FBS: fasting blood glucose
BMI: body mass index
CT: chest computed tomography
MRI: magnetic resonance imaging
rt-PA: intravenous recombinant tissue plasminogen activator
ICU: intensive care unit
AIS: acute ischemic stroke
FAST: F: facial drooping
A: arm weakness
S: speech difficulties
T: time to call for an emergency
